# Water Extract of *Fructus Hordei Germinatus* Shows Antihyperprolactinemia Activity via Dopamine D2 Receptor

**DOI:** 10.1155/2014/579054

**Published:** 2014-08-28

**Authors:** Xiong Wang, Li Ma, En-jing Zhang, Ji-li Zou, Hao Guo, Si-wei Peng, Jin-hu Wu

**Affiliations:** ^1^Department of Pharmacy, The Third Hospital of Wuhan, Wuhan, Hubei 430060, China; ^2^College of Pharmacy, Hubei University of Traditional Chinese Medicine, Wuhan, Hubei 430065, China

## Abstract

*Objective*. *Fructus Hordei Germinatus* is widely used in treating hyperprolactinemia (hyperPRL) as a kind of Chinese traditional herb in China. In this study, we investigated the anti-hyperPRL activity of water extract of *Fructus Hordei Germinatus* (WEFHG) and mechanism of action. *Methods*. Effect of WEFHG on serum prolactin (PRL), estradiol (E2), progesterone (P), follicle-stimulating hormone (FSH), luteinizing hormone (LH), and hypothalamus protein kinase A (PKA) and cyclic adenosine monophosphate (cAMP) levels of hyperPRL rats were investigated. And effect of WEFHG on PRL secretion, D2 receptors, and dopamine transporters (DAT) was studied in MMQ, GH3, and PC12 cells, respectively. *Results*. WEFHG reduced the secretion of PRL in hyperPRL rats effectively. In MMQ cell, treatment with WEFHG at 1–5 mg/mL significantly suppressed PRL secretion and synthesis. Consistent with a D2-action, WEFHG did not affect PRL in rat pituitary lactotropic tumor-derived GH3 cells that lack the D2 receptor expression but significantly increased the expression of D2 receptors and DAT in PC12 cells. In addition, WEFHG reduced the cAMP and PKA levels of hypothalamus in hyperPRL rats significantly. *Conclusions*. WEFHG showed anti-hyperPRL activity via dopamine D2 receptor, which was related to the second messenger cAMP and PKA.

## 1. Introduction

Hyperprolactinemia, which is one of the most common endocrine disorders of the hypothalamus-pituitary axis (PRL > 25 ng/mL) in young women, is associated with galactorrhea and ovulatory dysfunction that results in menstrual irregularities and barren [1]. HyperPRL can occur at any age, and the prevalence varies from 0.4% in the normal adult population to as high as 9–17% in women with menstrual problems such as amenorrhea or polycystic ovarian syndrome [2, 3]. Typical examples which induce hyperPRL are hypothalamus-pituitary lesions, pituitary tumor, severe liver or kidney disease, neuritis or irritations of the spinal cord, depression, or other physiological factors such as pregnancy and lactation [4, 5]. Galactorrhea is a common kind of female disease induced by hyperPRL. Synthetic drugs are used in treating them, but they always bring many side effects such as menstrual disorder and the relapse rate is very high. Bromocriptine and cabergoline are effective in curing hyperPRL, but 12% of patients can not endure bromocriptine [6], and the expense of them is very expensive [7].

Empirical evidence suggests that many herbal medicines possess the therapeutic potential to alleviate hyperPRL symptoms [8].* Fructus Hordei Germinatus* is a kind of herb germinating from barley, which was recorded to treat female galactorrhea in the ancient book 〈Zhonghuazi〉 of Song Dynasty [9]. In China,* F. H. Germinatus* is often used to treat galactorrhea effectively without toxic side effect [10, 11]. It was reported that* F. H. Germinatus* extract could decrease prolactin level in hyperPRL mice [12, 13].

In order to clarify the mechanisms underlying the anti-hyperPRL effects of WEFHG, we planned a direct experimental test. Most conventional anti-hyperPRL agents reduce PRL secretion through D2 receptor agonism in the hypothalamic neuroendocrine dopaminergic system, and cAMP/PKA signal transduction pathway is one of dopamine receptor classic pathways [14]. And other sex-steroids other than PRL and DAT are also involved in the pathophysiology of hyperPRL [15]. Therefore, we hypothesized that the therapeutic efficacy of WEFHG in alleviating hyperPRL could be attributed to modulation of dopamine D2 receptor. To test this hypothesis, we examined the effects of WEFHG on modulation of PRL, E2, P, FSH, and LH, PKA and cAMP levels in the rat model of hyperPRL, and D2 receptor- and DAT-mediated responses and PRL secretion in cell-culture systems.

## 2. Materials and Method

### 2.1. Preparation of WEFHG

The herbal samples of* F. H. Germinatus* were collected from Bozhou, Anhui, in July 2012. Taxonomic identification of the plant was performed by Professor Ke-li Chen of Hubei University of Chinese Medicine in China. The* F. H. Germinatus* (4.0 kg) was extracted with 40 L of filtered tap water at 100°C for 3 h. The water extract was filtered with standard sieves and then lyophilized. The lyophilized powder (yield: 3.4% of dry weight) was resuspended in distilled water and centrifuged at 10,000 ×g for 5 min to prepare WEFHG. After being filtered through a 0.2 *μ*m filter, WEFHG was used for* in vitro* and* in vivo* experiments.

### 2.2. *In Vivo* Experiment in Animal Model of HyperPRL

Female Wistar rats weighing 200–220 g were obtained from Hubei Center for Diseases Control and Prevention, Wuhan, Hubei. The animals had free access to food and water and were allowed to acclimatize for at least one week before use. All experiments were approved by the Animal Care and Use Committee and were carried in compliance with the Animal Welfare Act and the NIH guidelines (NIH publication number 80–23, revised 1996).

Rats were given intraperitoneal (i.p.) metoclopramide (MCP, 150 mg/kg daily), a dopamine inhibitor for 10 days to prepare experimental model of hyperPRL. The model has been widely used for investigation of hyperPRL [16]. 60 rats were divided into six groups of ten individuals: control, model group, model plus positive drug bromocriptine group, plus high dose of WEFHG (12.8 g/kg) group, plus middle dose of WEFHG (6.4 g/kg) group, and plus low dose of WEFHG (3.2 g/kg) group. Each dose was dissolved in a 2 mL water and administered gavage. The dosage was calculated from the daily human AFH clinical dosage based on body surface area. Control and model rats received 2 mL of water. All group mice were intragastrically administrated for 20 days. Blood samples were collected at the completion of experimental treatment and sera were separated for the measurement of PRL, E2, P, FSH, and LH.

### 2.3. *In Vitro* Experiments in Culture Cells

#### 2.3.1. Cell Lines and Culture

*In vitro* experiments, MMQ, GH3, and PC12 cell lines were used. Dose-dependent and time-course responses of PRL secretion and synthesis to WEFHG treatment were evaluated in MMQ cell line. GH3 cells were derived from rat pituitary lactotropic tumoral cells that lack D2 receptor expression. PC12 cells from rat pheochromocytoma abundantly express D2 receptors and DAT. Effects of WEFHG on these two dopamine mediators were further examined in PC12 cells.

MMQ, GH3, and PC12 cell lines were cultured in 65 cm^2^ flask, supplemented with 5% fetal calf serum (FBS), 11% heat-inactivated horse serum (HS), penicillin (100 IU/mL), and streptomycin (100 *μ*g/mL) under a humidified atmosphere containing 5% CO_2_ at 37°C. Replace the culture medium with fresh medium every two days. When the density reached 75% confluence, transfer culture cells to 35 mm diameter 6-well plates for experimental treatment.

#### 2.3.2. Experimental Design

MMQ cells were treated with WEFHG at concentrations of 0.5–4 mg/mL for 12–48 h. At different incubation time points, collect the culture medium for measuring PRL secretion. Cells were collected for determining cellular PRL synthesis. The optimal concentrations and treatment duration were then determined for subsequent experiments. GH3 and PC12 cells were treated with WEFHG at effective concentrations that had been examined in MMQ cells for 24 h. The GH3 cells and medium were collected for the measurement of PRL secretion and synthesis, respectively. PC12 cells were collected for detecting D2 receptor and DAT expressions. Two-way ANOVA was used for analyzing the effects of WEFHG on prolactin secretion versus time.

### 2.4. Biochemical Analyses

#### 2.4.1. Hormone Assay

PRL concentrations in the culture medium collected from MMQ and GH3 cells as well as PRL, E2, P, FSH, and LH concentrations in the rat sera were measured using enzyme-linked immunosorbent assay (ELISA) kits (Shenzhen Xin-Bo-Sheng Biological Technology Co., Ltd., China).

#### 2.4.2. Western Blotting

Expressions of intracellular PRL in MMQ and GH3 cells as well as D2 receptors and DAT in PC12 cells were determined by western blotting. Extract the cell proteins and determine the concentration by Bradford method. Proteins were separated by a 10% SDS-PAGE gel and transferred electrophoretically onto nitrocellulose membranes (Bio Basic, Inc.). Perform the immunodetection with the primary antibodies against PRL, D2 receptors, and DAT at a dilution of 1 : 1000 at 4°C overnight, followed by coincubation with horseradish peroxidase- (HRP-) conjugated secondary antibodies for 30 min at room temperature. Serve the primary antibody against glyceraldehyde 3-phosphate dehydrogenase (GAPDH) as a standard control for protein loading. Determine the chemiluminescence by ECL detection kits (Shang hai BestBio, China). The intensity of protein bands was quantified by scanning densitometry with Quantity One 4.5.0 software.

### 2.5. Measurement of Hypothalamus PKA and cAMP Levels

Rat brains were stripped and grinded into homogenate and then centrifugated for 15 min at 3000 r·min^−1^. The supernatant was used to measure PKA and cAMP levels with ELISA kits (Shenzhen Xin-Bo-Sheng Biological Technology Co., Ltd., China).

### 2.6. Statistical Analysis

One- or two-way variance analysis (ANOVA) was used to detect statistical significance, followed by* post hoc* multiple comparisons (Student-Newman-Keuls method). Data are expressed as mean ± S.E.M. Statistical significance was defined as *P* < 0.05.

## 3. Results

### 3.1. Effects of WEFHG on Sex Hormones in Rat Model of HyperPRL

**Figure 1:**
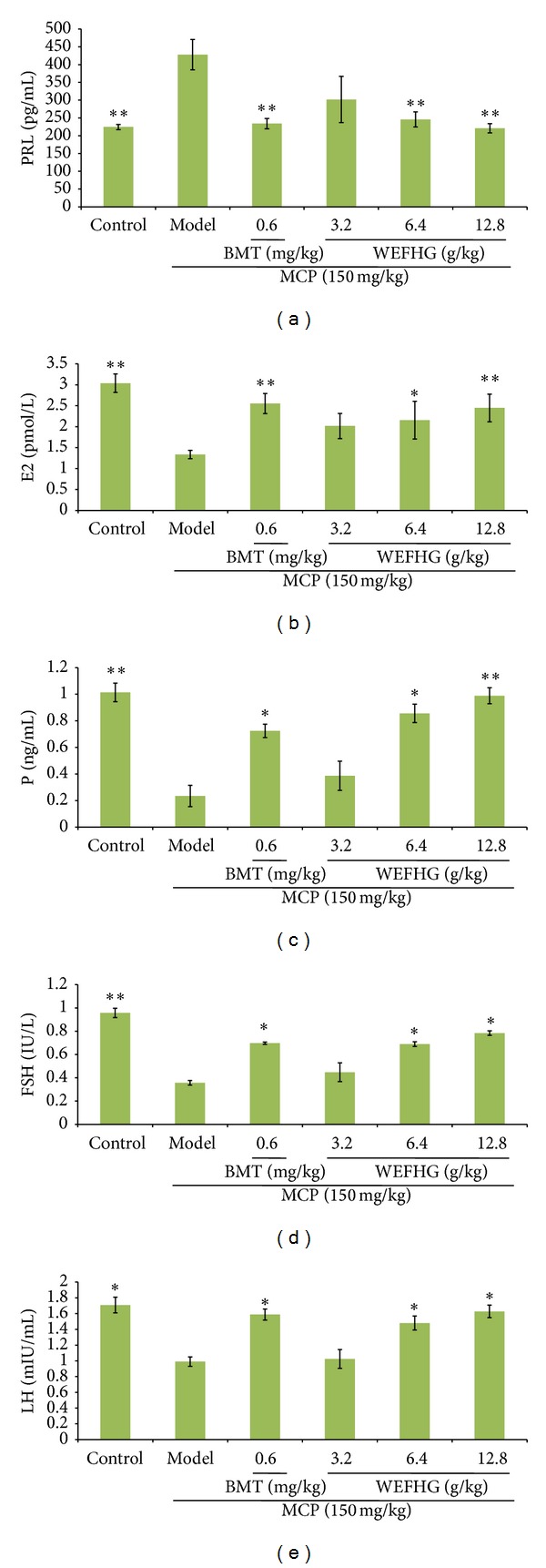
HyperPRL model of rat was prepared by injecting metoclopramide (MCP, 150 mg/kg) for 10 days, followed by treatment with or without bromocriptine (BMT) or WEFHG at different doses for 20 days. Untreated animals served as control. Serum PRL (a), E2 (b), P (c), FSH (d), and LH (e) were measured. Data are expressed as mean ± SEM (*n* = 10) and analyzed using one-way ANOVA. *P < 0.05 and **P < 0.01 versus model group.

HyperPRL inhibits oestrogen synthesis in the ovary and brings female hypogonadism, infertility, and amenorrhea [17]. Serum PRL (*P* < 0.01 versus control) concentrations increased significantly; E2, P, FSH (*P* < 0.01 versus control), and LH (*P* < 0.05 versus control) concentration decreased in hyperPRL model rats. Such an increased PRL was significantly attenuated by treatment with 0.6 mg/kg bromocriptine or 6.4 or 12.8 g/kg WEFHG after one month of administration (*P* < 0.01 versus the model group). Bromocriptine was effective in regulating E2 (*P* < 0.01 versus model), P (*P* < 0.05 versus model), FSH (*P* < 0.05 versus model), and LH (*P* < 0.05 versus model). And E2 (*P* < 0.01 versus model), P (*P* < 0.01 versus model), FSH (*P* < 0.05 versus model), and LH (*P* < 0.05 versus model) were regulated by 12.8 g/kg WEFHG obviously (Figure 1). The results suggest that WEFHG could inhibit the secretion of PRL and regulate other sex hormone levels in hyperPRL rats effectively.

### 3.2. Effects of WEFHG in Suppressing Hyperactive PRL in MMQ and GH3 Cells

**Figure 2:**
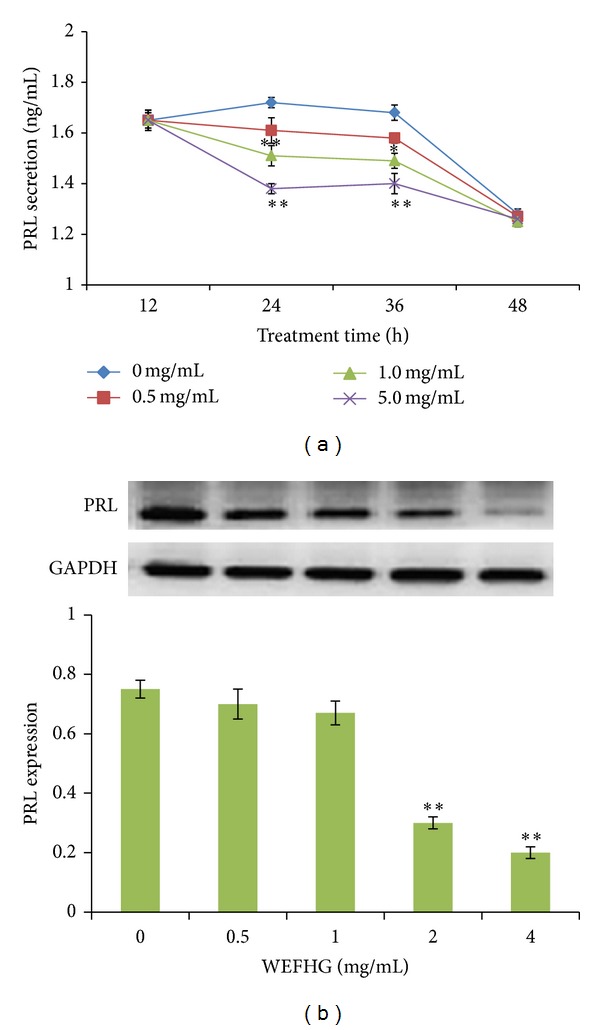
Time-course and dose-dependent effects of WEFHG in suppressing PRL secretion (a) and dose-dependent effects of WEFHG in inhibiting intracellular PRL expression (b) in MMQ cells. For the intracellular PRL expression, the cells were treated with different concentrations of WEFHG for 24 h. Data are expressed as mean ± SEM (*n* = 3) and analyzed using one- or two-way ANOVA. *P < 0.05 and **P < 0.01 versus 0 mg/mL group.

**Figure 3:**
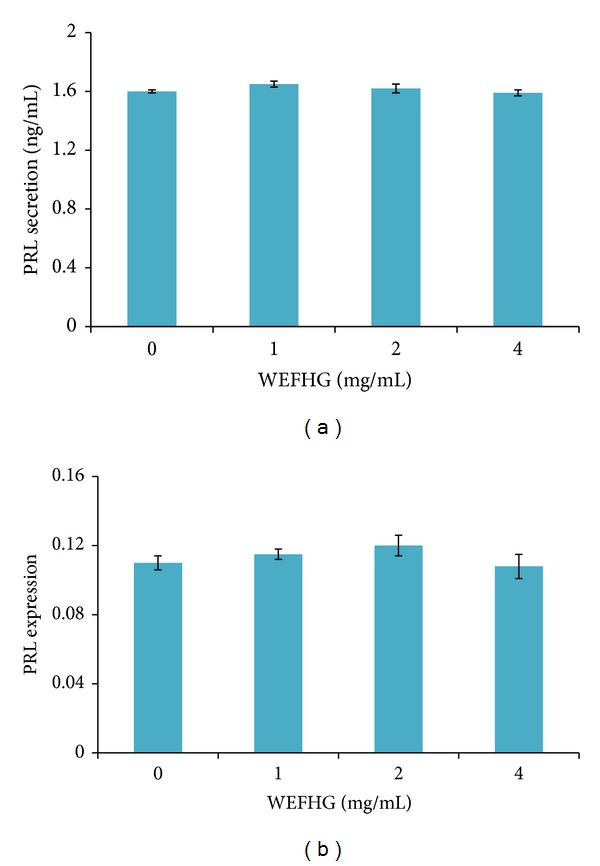
Effects of 24 hr WEFHG treatment on PRL secretion (a) and synthesis (b) in GH3 cells. Data are expressed as mean ± SEM (*n* = 3) and analyzed using one-way ANOVA. No significant differences were found in any multiple comparisons.

Two-way ANOVA analysis exhibited a significant interaction between time course and treatment group (*F* = 2.214, *P* = 0.050).* Post hoc* multiple comparison further revealed that 1 mg/mL and 5 mg/mL WEFHG treatment for 24 h and 36 h, but not 12 h and 48 h, produced a significant suppression of PRL concentration in the MMQ culture medium compared with controls (0 mg/mL) (*P* < 0.007) (Figure 2(a)). 24 hours were then chosen in further* in vitro* experiments. WEFHG treatment for 24 h also yielded a significant suppression of MMQ cellular PRL expression in a dose-dependent manner (*F* = 30.896, *P* < 0.001). Compared to controls, the significant suppression was observed in the higher two concentrations (2 and 4 mg/mL) (*P* < 0.003) (Figure 2(b)). But the same concentrations did not produce the significant effects on the medium concentration (*F* = 2.678, *P* = 0.113) and the cellular expression (*F* = 0.243, *P* = 0.874) in GH3 cells (Figure 3). These results suggest that WEFHG inhibited PRL secretion in hyperPRL rats dependent on dopamine D2 receptor.

### 3.3. Effects of WEFHG in Enhancing Dopamine Expression in PC12 Cells

**Figure 4:**
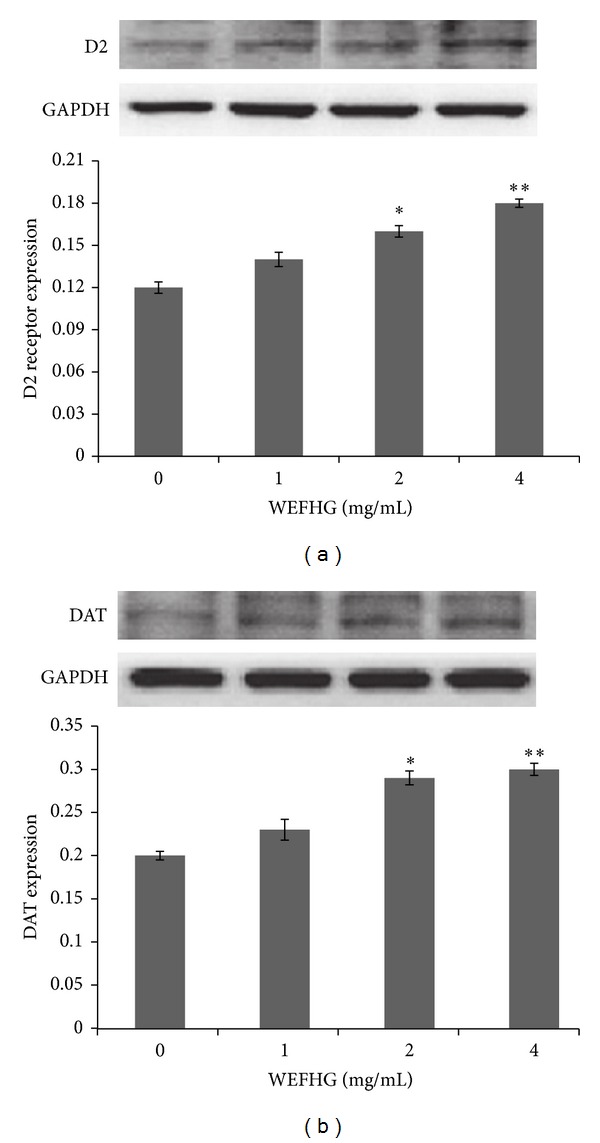
Effects of 24 h WEFHG treatment on the D2 receptor expressions (a) and DAT expressions (b). Data are expressed as mean ± SEM (*n* = 4) and analyzed using one-way ANOVA. *P < 0.05 and **P < 0.01 versus 0 mg/mL group.

PC12 cells were treated with 1–4 mg/mL concentrations of WEFHG for 24 h. One-way ANOVA revealed that WEFHG increased the expression of D2 receptors significantly (*F* = 6.682, *P* = 0.002) and DAT (*F* = 5.127, *P* = 0.014) (Figure 4). Multiple comparisons further exhibited that significant effects were attributed to concentrations of 2 mg/mL and 4 mg/mL compared to controls for D2 receptors (*P* < 0.015) and DAT (*P* < 0.028). These results suggest that D2 receptor and DAT played important roles in anti-hyperPRL activity of WEFHG. It has been well demonstrated that DAT participates in the physiological regulation of PRL and blockade of DAT inhibited PRL gene expression and secretion in female rats [15].

### 3.4. Effects of WEFHG on Hypothalamus PKA and cAMP Levels in Rat Model of HyperPRL

**Figure 5:**
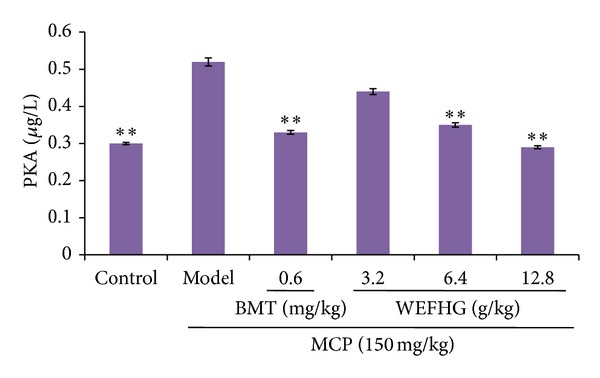
Hypothalamus PKA levels were measured. Data are expressed as mean ± SEM (*n* = 10) and analyzed using one-way ANOVA. *P < 0.05 and **P < 0.01 versus model group.

**Figure 6:**
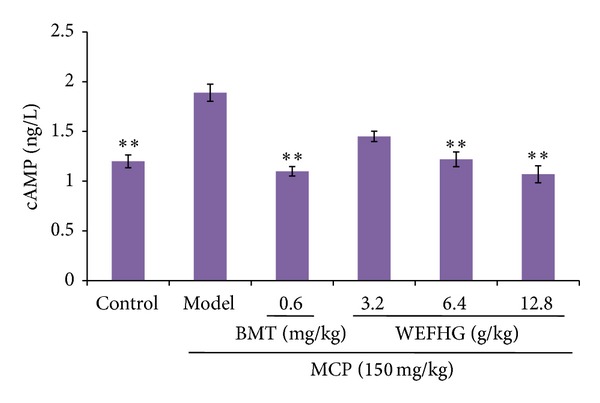
Hypothalamus cAMP levels were measured. Data are expressed as mean ± SEM (*n* = 10) and analyzed using one-way ANOVA. *P < 0.05 and **P < 0.01 versus model group.

Our study demonstrated that hypothalamus PKA (*P* < 0.01) and cAMP (*P* < 0.01) levels in rat model of hyperPRL increased significantly compared to control group. Such increased PKA and cAMP were significantly attenuated by treatment with 0.6 mg/kg bromocriptine or 6.4 or 12.8 g/kg WEFHG for 20 days (*P* < 0.01) (Figures 5 and 6). These results suggested that WEFHG showed anti-hyperPRL activity related to cAMP and PKA.

## 4. Discussion

Many previous studies support the use of* F*.* H*.* Germinatus* in traditional medicine for the treatment of hyperprolactinemia. In this paper, WEFHG reduced the secretion of PRL in hyperPRL rats effectively, and we analyzed the effect on MMQ, GH3, and PC12 cells of water extract of* Fructus Hordei Germinatus*, respectively.

MMQ cells, which express D2 receptor, are an exemplary model of hyperPRL derived from rat pituitary adenoma cells responsive to dopamine [18]. Dose-dependent and time-course responses of PRL secretion and synthesis to WEFHG treatment were evaluated in this cell line. In MMQ cell, treatment with WEFHG at 1–5 mg/mL significantly suppressed PRL secretion and synthesis. GH3 cells were derived from rat pituitary lactotropic tumoral cells that lack D2 receptor expression [19, 20], which were used to determine if deficiency of D2 receptors altered the suppression of WEFHG on hyperactive PRL. Consistent with a D2-action, WEFHG did not affect PRL in rat pituitary lactotropic tumor-derived GH3 cells that lack the D2 receptor expression.

These studies revealed that D2 receptor is necessary for anti-hyperPRL activity of WEFHG. PC12 cells from rat pheochromocytoma abundantly express D2 receptors and DAT [21, 22]. WEFHG significantly increased the expression of D2 receptors and DAT in PC12 cells. These suggested that D2 receptor and DAT all played important roles in anti-hyperPRL activity of WEFHG.

Dopamine receptors belong to the family of seven transmembrane domain G-protein coupled receptors (GPCR) [23–25]. Dopamine receptors D1 and D2 are classified into two subfamilies based on their differential effect on adenylyl cyclase. Classically, the functions of dopamine receptors have been associated with the regulation of adenylate cyclase-protein kinas A (cAMP-PKA) through G-protein-mediated signaling. Two classes of GPCR mediate dopamine functions; D1-like receptor subtypes (D1 and D5) couple mostly to Ga_s_ and stimulate the production of the second messenger cAMP and the activity of PKA. By contrast, D2-like subfamily (D2, D3, and D4) couple to Ga_i/o_ and regulate the production of cAMP negatively, thus resulting in a diminution of PKA activity [24–28]. The physiological and pathological roles of DR2 have been recognized in some organs such as brain and kidney [29, 30]. In the adenohypophysis, the predominant dopamine receptor is the D2 receptor [31]. Transfection of the dopamine D2 receptor into a pituitary cell line results in a decrease in intracellular cAMP and inhibits prolactin secretion when dopamine is added to the cell culture [32, 33]. Most conventional anti-hyperPRL agents reduce PRL secretion through D2 receptor agonism in the hypothalamic neuroendocrine dopaminergic system, and cAMP/PKA signal transduction pathway is one of dopamine receptor classic pathways. In our study, WEFHG inhibited PRL secretion dependent on dopamine D2 receptor in MMQ cell and reduced the PRL, cAMP, and PKA levels of hypothalamus in hyperPRL rats significantly. These suggested that WEFHG showed anti-hyperPRL activity via dopamine D2 receptor, which was related to the second messenger cAMP and PKA. Further study about whether WEFHG showed anti-hyperPRL activity via cAMP/PKA signal transduction pathway was needed.

## 5. Conclusions

From above studies, it showed that WEFHG inhibited the secretion of PRL and reduced the levels of hypothalamus PKA and cAMP in hyperPRL rats effectively. D2 receptor and DAT played important roles in anti-hyperPRL activity of WEFHG. Our study demonstrated that WEFHG showed anti-hyperPRL activity via dopamine D2 receptor, which was related to the second messenger cAMP and PKA.
